# HGF/c-met/Stat3 signaling during skin tumor cell invasion: indications for a positive feedback loop

**DOI:** 10.1186/1471-2407-11-180

**Published:** 2011-05-19

**Authors:** Zanobia A Syed, Weihong Yin, Kendall Hughes, Jennifer N Gill, Runhua Shi, John L Clifford

**Affiliations:** 1 Department of Biochemistry and Molecular Biology, Louisiana State University Health Sciences Center-Shreveport and Feist Weiller Cancer Center, 1501 Kings Hwy, Shreveport, Louisiana, 71103, USA; 2 Department of Medicine, Louisiana State University Health Sciences Center-Shreveport and Feist Weiller Cancer Center, 1501 Kings Hwy, Shreveport, Louisiana, 71103, USA; 3 Wake Forest Institute for Regenerative Medicine, Wake Forest University School of Medicine, Medical Center Boulevard, Winston-Salem, NC 27157, USA

## Abstract

**Background:**

Stat3 is a cytokine- and growth factor-inducible transcription factor that regulates cell motility, migration, and invasion under normal and pathological situations, making it a promising target for cancer therapeutics. The hepatocyte growth factor (HGF)/c-met receptor tyrosine kinase signaling pathway is responsible for stimulation of cell motility and invasion, and Stat3 is responsible for at least part of the c-met signal.

**Methods:**

We have stably transfected a human squamous cell carcinoma (SCC) cell line (SRB12-p9) to force the expression of a dominant negative form of Stat3 (S3DN), which we have previously shown to suppress Stat3 activity. The *in vitro *and *in vivo *malignant behavior of the S3DN cells was compared to parental and vector transfected controls.

**Results:**

Suppression of Stat3 activity impaired the ability of the S3DN cells to scatter upon stimulation with HGF (c-met ligand), enhanced their adhesion, and diminished their capacity to invade *in vitro *and *in vivo*. Surprisingly, S3DN cells also showed suppressed HGF-induced activation of c-met, and had nearly undetectable basal c-met activity, as revealed by a phospho-specific c-met antibody. In addition, we showed that there is a strong membrane specific localization of phospho-Stat3 in the wild type (WT) and vector transfected control (NEO4) SRB12-p9 cells, which is lost in the S3DN cells. Finally, co-immunoprecipitation experiments revealed that S3DN interfered with Stat3/c-met interaction.

**Conclusion:**

These studies are the first confirm that interference with the HGF/c-met/Stat3 signaling pathway can block tumor cell invasion in an *in vivo *model. We also provide novel evidence for a possible positive feedback loop whereby Stat3 can activate c-met, and we correlate membrane localization of phospho-Stat3 with invasion *in vivo*.

## Background

Signal transducer and activator of transcription (Stat) proteins are a family of transcription factors that are activated by phosphorylation of a conserved tyrosine residue in response to a host of growth factors and cytokines. Phosphorylated Stat dimers translocate into the nucleus to activate of several target genes that are involved in diverse cellular processes such as cytokine signaling [1], cell proliferation and development [2], abnormal tumorigenesis [3-5], and suppression of the immune response in the tumor microenvironment [6]. Stat3, one of seven members of the Stat family, has been most strongly implicated in tumorigenesis [3-5]. Stat3 regulated genes include cyclin D1 [7] and c-myc [8], which are involved in cell proliferation; Bcl-X_L _[9], survivin [10], and Bcl-2 [11], which mediate apoptosis; several matrix metalloproteases involved in invasion [12-14]; and growth factors and cytokines such as VEGF [15] and HGF [16]. Up- or down-regulation of many of these genes has obvious implications in the development of cancer. Constitutive activation of Stat3 has been observed in a number of human cancers and cancer cell lines [3]. Given that no naturally occurring Stat3 mutations that result in constitutive activity have been identified, the persistent Stat3 activation in tumors is likely due to a differences in expression or activity of proteins that regulate Stat3 or signaling molecules involved in the Stat3 pathway. Potential candidates include suppressor of cytokine signaling (SOCS), a negative regulator of cytokine signaling that is silenced by methylation in some tumors [17,18] and various receptor tyrosine kinases such as EGFR and c-met that are activated in cancers [19,20]. More recent evidence indicates that Stat3 may be a required for the maintenance of stem cell-like characteristics of glioblastoma stem cells [21]. Given the central role of stat3 in positively effecting multiple biological processes involved in malignant cell behavior, extensive effort has been made to target Stat3 and suppress its activity in cancer cells [22]. One recent preclinical study using myeloid and B cell-specific targeting of Stat3 by siRNA showed that silencing Stat3 expression lead to a strong antitumor immune response [23].

Stat3 has a critical function in the development of skin cancer [24]. Using both skin-specific Stat3 knockout models [25,26], and a skin specific Stat3 gain of function transgenic model (K5.Stat3C mice) [27], we have shown in collaboration with the DiGiovanni laboratory that Stat3 is indispensable for the initiation, promotion and malignant progression stages of skin carcinogenesis. More recently it has been shown, using skin stem cell-specific knockout of Stat3, that Stat3 is required for survival of skin stem cells during tumor initiation in the mouse skin 2-stage chemical carcinogenesis protocol, and that it is indeed these stem cells that form the initiated cell population that eventually gives rise to tumors [28]. These results are reviewed in [29,30].

Malignant progression and tumor cell invasion are often the result of uncontrolled cell motility and adhesion. There is considerable evidence for a role for Stat3 signaling in cell migration and invasion under normal and pathological situations. Overexpression and activity of Stat3 has been linked to the invasion and metastasis of several cancers in humans, including cutaneous squamous cell carcinoma (SCC) [31,32], colorectal adenocarcinoma [33], and melanoma [14]. Stat3 activation is required for induction of genes encoding matrix metalloproteases-1, 2, and 9 (MMP-1, MMP-2, and MMP-9) [12-14], as well as several other genes important in the metastatic cascade. In addition, Stat3-deficient keratinocytes are unable to migrate, partly due to deregulated p130^CAS ^phosphorylation [34], a protein which is involved in the formation of focal adhesion complexes and reorganization of the cytoskeleton. These studies, as well as our studies with mouse skin-specific Stat3 gain of function and knockout models [29,30], suggest that Stat3 activation plays a critical role in the invasion and metastasis of carcinomas.

Scatter factor, or hepatocyte growth factor (HGF), can regulate cell survival, growth, migration, and angiogenesis upon binding its cell surface receptor, c-met [35]. c-met is often constitutively active in human tumors [36-38], and HGF/c-met signaling is at least partially mediated by Stat3 [20,39]. In particular, Stat3 is transiently phosphorylated after HGF treatment, likely mediated by Stat3/c-met direct or indirect interaction [39]. Stat3 was coimmunoprecipitated with phosphorylated c-met from intact cells, and the specificity of this interaction was demonstrated in *in vitro *competition experiments with peptides mimicking the c-met docking sequence and Stat3 phosphorylation site [39]. Activated Stat3 mediates signals downstream of the HGF/c-met pathway [20], and Stat3 has been shown to be essential for HGF-induced morphogenesis and for invasive behavior driven by c-met in both fibroblasts and breast carcinoma cells [39-41]. A Stat3 binding site has been identified in the HGF promoter and this site is required for Stat3-mediated activation of HGF transcription in breast epithelial cells [16,42]. Overall, these observations suggest that positive feedback signals in the HGF/c-met/Stat3 signaling pathway could contribute to tumorigenesis.

In order to investigate the role of Stat3 in the induction of cell motility and invasion in greater detail, we have employed a cell culture model in which a dominant-negative form of Stat3 was expressed in a tumorigenic human skin SCC cell line, SRB12-p9 [43]. Stat3α, the full-length form of Stat3, is abundant and is constitutively phosphorylated (active) in these cells. It has been reported that Stat3β, a naturally occurring Stat3 splice variant that lacks the C-terminal transcriptional activation domain, can act in a dominant-negative fashion to inhibit the transcriptional activity of Stat3α [44,45]. It was subsequently shown that mutating the critical tyrosine 705 phosphorylation site to phenylalanine generated a form of Stat3β (Stat3β-Y705F) that could block DNA binding by all endogenous forms of Stat3 [46]. In this study we compare the malignant characteristics of SRB12-p9 cells with that of stably transfected clones that express FLAG-tagged Stat3β-Y705F (S3DN cells), which have suppressed Stat3 activity [43]. We confirm for the first time that interference with the HGF/c-met/Stat3 signaling pathway can block tumor cell invasion in an *in vivo *model, and we present evidence for a positive feedback loop between Stat3 and c-met. Finally we correlate membrane localization of phospho-Stat3 with invasion *in vivo*.

## Methods

### Cell culture

The human skin SCC cell line SRB12-p9 was derived by single cell cloning from SRB-12 cells (a gift from Dr. Janet Price, Department of Cancer Biology, University of Texas M.D. Anderson Cancer Center). Cells were cultured in a humidified atmosphere at 5% CO_2_, in Dulbecco's Modified Eagle's Media-F12 supplemented with 10% fetal calf serum. Generation of the stable S3DN expressing SRB12-p9 cell lines and vector control cell lines was previously described [43].

### Invasion and scattering assays

8 μm pore-sized membrane inserts for 12 well plates were coated with 50 μL of 1:25 diluted Matrigel basement membrane matrix (Becton Dickinson) following the manufacturer's protocol. 25,000 WT, NEO4 and S3DN cells were seeded in triplicate onto inserts and incubated for 10 hours at 37°C in 5% CO_2_. No chemo-attractant or serum gradient was used. After incubation, membranes were fixed with 4% paraformaldehyde for 20 minutes and stained with hematoxylin. Cells that migrated through the Matrigel to the bottom of the insert were photographed on a Nikon TE300 microscope and counted. For the scattering assay, cells growing in 10% FCS-containing media were treated with 100 ng/ml HGF for 24 or 48 hours and photographed with phase contrast at 100× magnification on a Nikon TE300 microscope. Percent cell scattering was determined by counting the total number of cells in a field (T), and the number of cells which were visually determined to have no cell-cell contacts (M) in the field, and applying the following formula: percent scattering = (M/T) × 100%. At least 6 fields were counted for each treatment group and the fields were marked on the bottom of the dish so that they could be located at the 24 and 48 hour time points.

### Western blotting

Cells were lysed and tumors homogenized on ice in RIPA lysis buffer (150 mM NaCl, 50 mM Tris-HCl pH 7.5, 1 mM EDTA, 1% NP-40, 1 mM PMSF, 1 mM Na-orthovanadate) supplemented with 40 μl complete protease inhibitor cocktail (Roche) according to manufacturer-provided instructions. Extracted protein was quantified using the BioRad Protein Assay kit. Proteins were separated by SDS acrylamide (4-20%) gel electrophoresis and transferred to nitrocellulose membranes (BioRad). Blots were blocked with 5% BSA for 1 hour at room temperature, followed by incubation overnight at 4°C with antibodies against phosphorylated forms of c-met (Tyr1234-1235) and Stat3 (Tyr705), total Stat3 (Cell Signaling Technology), total c-met and b-actin (Santa Cruz), and MMP-2 and MMP-9 (Chemicon). Blots were washed with TBS/0.1% Tween 20 and incubated with a horseradish peroxidase-conjugated secondary antibody for 1 hour at room temperature, followed by an additional 3 washes with TBS/0.1% Tween 20. Chemiluminescence detection was performed according to the manufacturer's instructions (Millipore) followed by exposure to X-ray film or using the Chemidoc gel documentation system (BioRad).

### Immunoprecipitation

After overnight incubation in serum free media, cells were treated with 100 ng/ml HGF for 30 minutes prior to harvest, and lysed with the following immunoprecipitation buffer: 1% NP-40 in PBS with 40 μl complete protease inhibitor cocktail (Roche), 2 mM Na-orthovanadate, 10 μM lactacystin, 10 mM NaF. Cell lysates (500 μg for each sample) were rocked at 4°C for 1 hour, followed by centrifugation for 5 minutes to pellet cellular debris. Supernatants were collected and samples were precleared with Protein A/G Plus-Agrarose (Santa Cruz) and normal IgG (Santa Cruz) for 1 hour at 4°C, followed by centrifugation for 5 minutes at 20,000 g. Total Stat3 (Cell Signaling Technology) and c-met (Santa Cruz) antibodies were added at a dilution of 1:1000 to a total volume of 1 ml, and immunoprecipitations were performed at 4°C overnight with constant rocking. 20 μl of Protein A/G Plus-Agarose (Santa Cruz) beads were added, and the solution was allowed to rock for 4 hours at 4°C. Reactions were washed 3 times using immunoprecipitation buffer and then brought up in 6× Laemli buffer for protein electrophoresis and Western blot analysis.

### Mouse xenograft model

Groups of 6-7 week old female athymic NCR Nu/Nu (nude) and SCID/bg mice were housed in a temperature and humidity controlled Association for Assessment and Accreditation of Laboratory Animal Care facility with a 12 hour light/dark cycle. All procedures were approved by the LSUHSC Institutional Animal Care and Use Committee in accordance with NIH guidelines. Mice were maintained on LM-485 diet (Harlan Teklad) and allowed access to food and water *ad libitum*. Mice were injected subcutaneously in the interscapular region with 1 × 10^6 ^cells in 0.1 ml PBS. The growth of the subcutaneous tumors was evaluated twice weekly by caliper. Animals were sacrificed and tumors were excised at the end of the experiment (21 days after cell injection) and fixed in 4% paraformaldehyde for histological observation, western blotting, and immunohistochemistry.

### Immunohistochemical examination of tumors

Tumors were isolated and fixed in formalin and embedded in paraffin prior to sectioning. Paraffin sections of 4 μm were deparaffinized in xylene, 3 × 7 minutes, and rehydrated by stepwise washes in decreasing ethanol/H_2_O ratio (100% to 50%, followed by soaking in water). Sections were either stained with hematoxylin and eosin (H&E) or boiled for 1 minute for antigen retrieval. For immunohistochemical staining, sections were incubated in Superblock (Pierce) blocking reagent for 1 hour at room temperature. After washing 3× in PBS, slides were incubated for overnight at 4°C with antibodies against p-c-met (Tyr1234-1235), p-Stat3 (Tyr705), and total Stat3 (Cell Signaling Technology), total c-met (Santa Cruz), and MMP-2 and MMP-9 (Chemicon). Slides were photographed on a Nikon TE300 fluorescence microscope under oil immersion, at 600× magnification, with a CCD camera (Roper Scientific). Images were processed with IPLabs v3.55 software (Scanalytics).

### Statistical analysis

The statistical significance between experimental values was assessed by Student's T test. A P value of < 0.05 was considered as statistically significant.

## Results

### Expression of S3DN suppresses the invasion and HGF-induced scattering of SRB12-p9 cells

**Figure 1:**
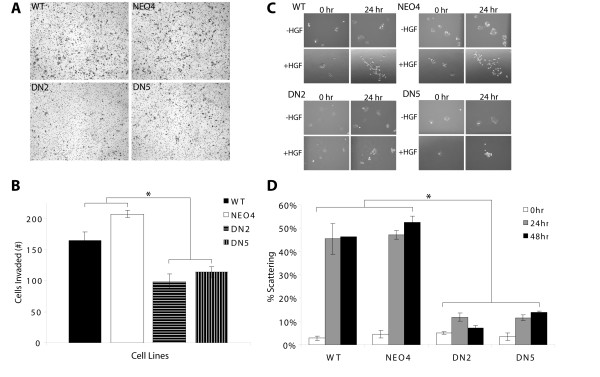
**Invasion and scattering of P9WT and S3DN cells in vitro**. (A) 25,000 cells cultured in 10% FCS containing media were plated on Matrigel-coated inserts and allowed to invade. Invaded cells were fixed, stained with hematoxylin, and counted. Values represent mean number of cells for triplicate cultures. (B) Quantification of invaded cells for each cell line. Values shown are +/- SEM and P < .05 by Student's T test for WT/S3DN and NEO4/S3DN comparisons indicated by *. (C) Cells growing in 10% FCS-containing media were treated with 100 ng/ml HGF for 24 or 48 hours as indicated and photographed with phase contrast at 100× magnification. (D) Quantification of scattered cells expressed as percent (of total cells) scattered per well. P < .05 by Student's T test for WT/S3DN and NEO4/S3DN comparisons indicated by *.

In order to assess the role of Stat3 signaling in the invasive potential of malignant SCC cells, we analyzed the parental SRB12-p9 wild type (WT) and empty vector transfected controls (NEO4), and two independent S3DN cell lines (DN2 and DN5), in an *in vitro *invasion assay. Cells were seeded onto Matrigel-coated inserts and allowed to invade for 10 hours. Expression of S3DN suppressed the ability of the DN2 and DN5 cells to invade the Matrigel barrier (Figure 1A). DN2 and DN5 cells showed reduced invasion, as compared to the WT and NEO4 cells, with an average of 99 and 114 cells per field migrating for the DN2 and DN5 cells, as compared to 165 and 207 cells for the WT and NEO4 cell lines, respectively (Figure 1B). The number of invading S3DN cells was reduced by 30-50% of that observed for WT and NEO4. It is unlikely that these effects are due to differences in cell survival or proliferation due to the relatively short time course of the experiment and to our previous studies that showed that the doubling time and basal rate of apoptosis are the same for S3DN and WT cells under normal culture conditions, as in this assay [43]. It is important to note that no chemo-attractant or serum gradient was used to enhance invasion in this experiment.

Because HGF has potent effects on the motility of keratinocytes, and since dermal fibroblasts produce HGF in the skin [47], we decided to examine the role of Stat3 signaling in HGF-induced motility. Cells were allowed to grow into colonies of 8-12 cells for several days, and then treated with 100 ng/mL human recombinant HGF (R&D Systems) for 24 and 48 hours. Motility was measured as the number of cells in a colony lacking cell-cell contact with their neighbors. WT and NEO4 cells responded to HGF treatment with nearly 50% of cells becoming motile by 24 hours and maintained that status up to 48 hours later (Figure 1C, D). However, DN2 and DN5 cells responded minimally to HGF treatment, with only 11-12% of total S3DN cells scattered at the 24 hour time point (Figure 1D).

### Suppression of Stat3 reduces activity of c-met in vitro: evidence suggesting a positive feedback loop

**Figure 2:**
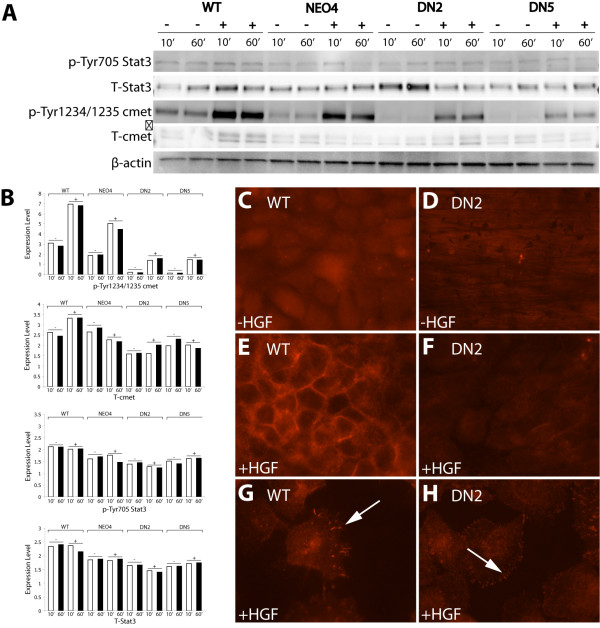
**Analysis of HGF-induced c-met activity in cell lines**. (A) WT, NEO4 and S3DN cells were stimulated with 100 ng/mL HGF for 10 and 60 minutes. Lysates were resolved on a 4-20% SDS polyacrylamide gel, transferred to nitrocellulose, and probed sequentially with antibodies for p-Stat3, total Stat3, p-c-met, total c-met, and β-actin. (B) Bar graph of western blot quantification. Values indicate the scanned signals normalized to the β-actin signal. (-) and (+) correspond to without and with HGF treatment, respectively. (C) WT & DN2 cells were stimulated with 100 ng/mL HGF for 30 minutes and probed with a p-c-met antibody, followed by an Alexa-546 tagged secondary antibody. Panels photographed at 600×. Arrows in C, lower panels indicate focal adhesion staining.

To better understand the molecular mechanisms by which suppression of Stat3 signaling can inhibit HGF-induced motility, the levels of expression and activity of c-met was determined for WT, NEO4 and S3DN cells. Cells were treated with 100ng/ml HGF for 10 and 60 minutes and harvested after each time point. Western blot analysis of cell lysates with phospho-specific and total c-met antibodies revealed a decrease in the level of phospho-c-met in DN2 and DN5 cells compared to WT and NEO4 cells, under both untreated and HGF treated conditions (Figure 2A, B). The reduced activity of c-met under unstimulated conditions in the S3DN cells is likely a result of the suppression of Stat3 signaling, suggesting a positive feedback effect of Stat3 on c-met. With HGF stimulation, the S3DN cells were unable to induce activation of c-met to the same level as the WT and NEO4 cells (Figure 2A, B). In fact, the sustained activity of c-met observed in the S3DN cells at 60 minutes is similar to that observed in the unstimulated WT and NEO4 cells. These results were further confirmed by immunofluorescence analysis of WT and DN2 cells grown in culture. The level of phospho-c-met found in DN2 cells is diminished as compared to the WT cells, even in the presence of HGF (Figure 2, compare WT panels C, E and G to DN2 panels D, F and H). We observe intense membrane localization of phospho-c-met in WT cells treated with HGF that is absent in the DN2 cells. Staining in cells growing at a lower confluency shows a staining pattern consistent with localization in focal adhesions in both cell lines. However, phospho-c-met specific staining is reduced in the focal adhesions of DN2 cells (Figure 2G and 2H). No stimulation of Stat3 phosphorylation above baseline levels was observed with HGF treatment (Figure 2A, top panel). This finding is not unexpected considering our previous results showing that both WT and S3DN cells have similarly high levels of constitutive Stat3 phosphorylation when grown in 10% FCS [43]. We also showed in that study that S3DN expression, while it did not suppress Stat3 phosphorylation, did reduce Stat3 DNA binding, indicating that Stat3 signaling was in fact impaired in the S3DN cells [43].

### S3DN inhibits the invasive potential of SCC cells in vivo

**Figure 3:**
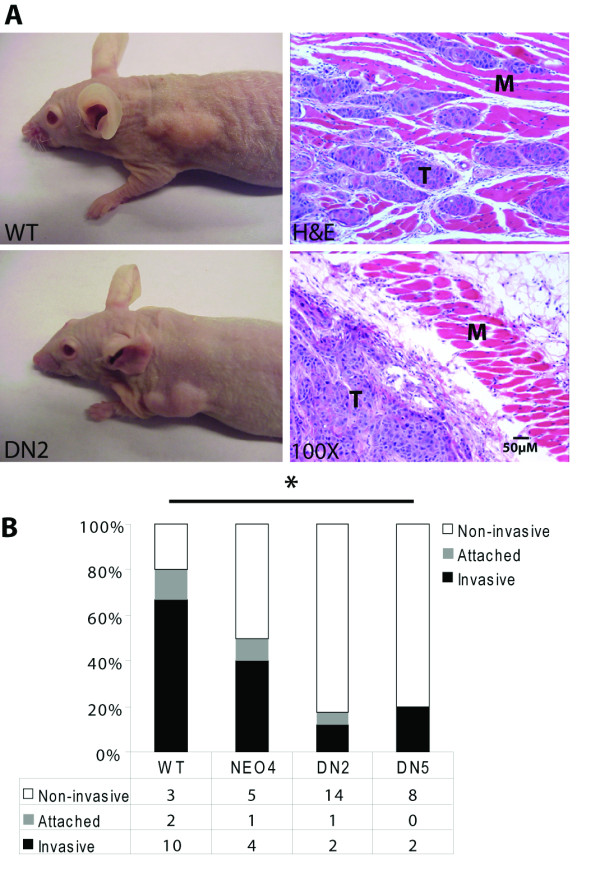
***In vivo *tumorigenicity potential of P9WT and S3DN cells**. (A) Nude and SCID mice were injected with WT and DN2 cells and tumors were assessed by immunohistochemical analysis. H&E stained paraffin sections of tumors generated from WT and DN2 cells were photographed at 100×. T (tumor) and M (muscle) indicate invasive growth of P9WT cells. (B) Tumor invasion bar graph. Tumors were designated as non-invasive, attached, or invasive and expressed as a percent of total tumors. * The 2-sided probability (p) ≤ 0.0006 comparing the tumor phenotype distribution of WT and NEO cells combined, with that of DN2 and DN5 cells combined.

To compare the *in vivo *tumorigenic potential of WT, NEO4 and S3DN cells, we used two immuno-compromised mouse tumorigenicity model systems. Both nude and SCID mice were injected subcutaneously on their dorsal side with 1 × 10^6 ^cells suspended in 100 μl of sterile PBS. Interestingly, expression of S3DN did not reduce total tumor volume, nor did it affect tumor growth kinetics, as had been predicted (Figure 3A, left panels and data not shown). However, closer examination of the tumors upon harvest revealed differences in tumor behavior that we segregated into three groups: non-invasive, for tumors that were encapsulated and easily removed from the body wall; attached, for tumors that had some superficial growth onto the muscle of the body cavity; and invasive, for tumors that showed complete invasion into the muscle and often had to be excised with muscle or bone together. Macroscopic examination revealed that the WT and NEO4 tumors were more often strongly attached to the musculature of the body wall, and in many cases were found to be invading into the muscle (Figure 3A, right panels, 3B). This was in contrast to the S3DN tumor group in which most tumors where either well encapsulated or easily removed from the underlying muscle (Figure 3A, lower right, 3B). In fact, only 18-20% of the tumors arising from the S3DN cells grew attached to the musculature or invaded into the body cavity, compared to 50-80% of the WT and NEO4 tumors (Figure 3B). The distribution of tumor phenotypes comparing WT to NEO4 cells was not statistically significantly different, with the 2-sided probability, p ≤ 0.261, using the Fisher's exact test. Similarly, the tumor phenotype distribution comparing DN2 to DN5 cells was not different (p ≤ 0.764). When comparing the tumor phenotype distribution of WT and NEO cells combined, with that of DN2 and DN5 cells combined, the difference was highly statistically significant (p ≤ 0.0006). There were no differences in the efficiency of tumor establishment, with all cell lines producing single tumors in the mice.

### Expression of S3DN alters the localization of active Stat3 in the cell

**Figure 4:**
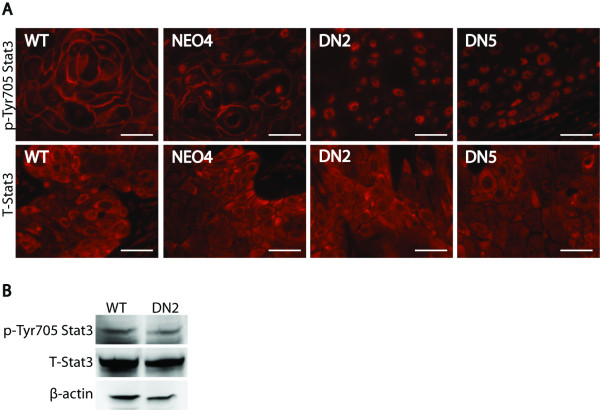
**Immunohistochemical staining of p-Tyr705 Stat3 and total Stat3 (T-Stat3) and localization in WT, NEO4 and S3DN tumors**. (A) Paraffin sections of tumors in SCID mice were probed with p-Tyr705 Stat3 and T-Stat3 antibodies, followed by an Alexa 546-labeled secondary antibody. Panels were photographed at 600×. (B) Lysates pooled from WT and DN2 tumors were resolved on a 4-20% SDS polyacrylamide gel, transferred to nitrocellulose, and probed with antibodies for p-Tyr705 Stat3, T-Stat3, and β-actin. Scale bar: 50 μM.

Since S3DN expression correlated with suppression of tumor invasion, we sought to determine whether it altered the levels or subcellular localization of active Stat3 in tumors. Stat3 activity is maintained in tumors arising from WT, NEO4 and S3DN cells, as determined by immunohistochemical staining with a Tyr705 phosphorylation site-specific antibody for Stat3 (Figure 4A, upper panels). There was also no observable difference in total Stat3 expression between WT, NEO4 and S3DN tumors, where the majority of bulk protein expression was observed in the cytoplasm (Figure 4A, lower panels, compare WT and NEO4 to DN2 and DN5). However, the subcellular localization of active Stat3 differed between the two groups of tumors. The DN2 and DN5 tumors expressed pTyr705 Stat3 primarily in the nuclei, which is the expected localization for activated Stat3 (Figure 4A, DN2 and DN5 panels). The WT and NEO4 tumors had nuclear expression of p-Tyr705 Stat3, but also showed strong staining localized to the cell membrane, particularly in the larger cells in squamous centers (Figure 4A, upper panels, compare WT and NEO4 to DN2 and DN5). This finding is consistent with our recent results in human skin SCC, where we found both prominent nuclear staining of p-Tyr705 Stat3 as well as membrane-specific staining in centers of squamous differentiation [48]. Total levels of Stat3 activity and expression were determined by Western blot analysis of tumor lysates. We found that, like the immunostaining results, there was no quantitative difference in the levels of either phospho-Stat3 (p-Tyr705 Stat3), which detects both the Stat3α and Stat3β bands, or total Stat3 expression (T-Stat3) (Figure 4B). We note that in spite of the lack of reduction of nuclear phospho-Stat3 in the S3DN cells, Stat3 activity in these cells is reduced. We previously showed that S3DN cells have reduced Stat3 DNA binding as measured by EMSA [43], verifying the suppressive effect of S3DN expression on Stat3 activity.

### S3DN reduces expression of MMP-2 and MMP-9, and inhibits c-met phosphorylation *in vivo*

**Figure 5:**
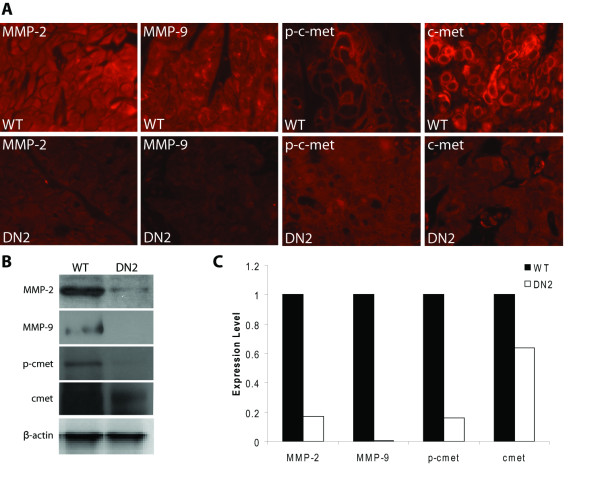
**Expression of tumor markers in WT and DN2 tumors**. (A) Paraffin sections of tumors generated from WT and DN2 cells were probed with MMP-2, MMP-9, p-c-met, and total c-met antibodies, followed by an Alexa 546-labeled secondary antibody. Panels were photographed at 600×. (B) Lysates generated from WT and DN2 tumors were resolved on a 4-20% SDS polyacrylamide gel, transferred to nitrocellulose, and probed sequentially with antibodies for p-c-met, total c-met, MMP-2, MMP-9, and β-actin. (C) Bar graph of western blot quantification.

We next determined the expression levels of MMP-2 and MMP-9, two key matrix metalloproteases involved in the degradation of the basement membrane, and frequently upregulated in invading tumor cells. Both MMP-2 and MMP-9 are transcriptional targets of Stat3 [12,14]. Immunostaining and Western blotting revealed that MMP-2 and MMP-9 expression was reduced in DN2 tumors compared to WT tumors, (Figure 5A, compare upper left panels lower left panels, 5B,C). Diminished matrix metalloprotease expression is one of the likely causes of the reduction in observed invasion in S3DN tumors. Gelatin zymography of conditioned media harvested from WT, NEO4 and S3DN cells also indicated that MMP-2 and MMP-9 activity is reduced in S3DN cells (data not shown).

Immunostaining and Western blot analysis of tumors for expression of phospho-c-met agreed with our *in vitro *results (Figure 2), where we also noted reduced phospho-c-met expression in DN2 tumors compared to WT (Figure 5, compare p-c-met panel to lower p-c-met panel, 5B,C). In fact, the intense membrane-specific localization, typical of activated c-met, that was observed in the WT tumors was absent in the DN2 tumors. Unexpectedly, we found that unlike in S3DN cells grown in culture, total c-met levels were diminished in DN2 tumors compared to WT (Figure 5A, far right panels, 5B,C).

### S3DN interferes with c-met/Stat3 interaction

**Figure 6:**
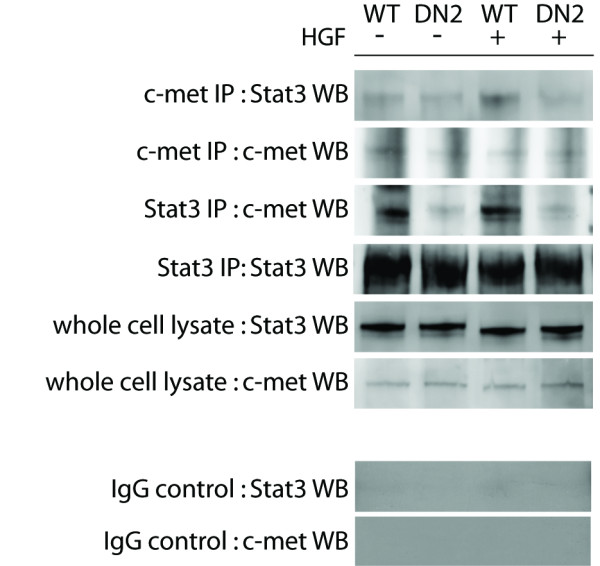
**Coimmunoprecipitation of Stat3/c-met**. WT and DN2 cells treated with 100 ng/ml HGF for 30 minutes and untreated controls were immunoprecipitated with total c-met and Stat3 antibodies. Western blot analysis of immunoprecipitates with reciprocal antibodies shows interaction between Stat3 and c-met, which is diminished in DN2 cells. Whole cell lysates probed with total Stat3 and c-met antibodies shown as loading control. IgG controls indicate the use of rabbit IgG instead of antibodies to c-met or Stat3 for the immunoprecipitations.

In an attempt to better understand the c-met/Stat3 interaction in this model, we analyzed the binding interaction between c-met and Stat3 in S3DN cells. It is possible that the reduced basal and HGF-inducible c-met activity in S3DN cells correlates with reduced c-met/Stat3 interaction. We used antibodies for total c-met and Stat3 for reciprocal immunoprecipitation experiments with WT and DN2 cells treated with HGF. We detected an interaction between c-met and Stat3, which was enhanced with HGF treatment in the WT but not DN2 cells (Figure 6, upper panel WT lanes). This level of interaction was lower in the DN2 cells compared to WT (Figure 6, top panel and third panel, compare WT lanes to DN2). These results suggest that a possible HGF/c-met/Stat3 positive feedback loop that is hyperstimulated in WT cells is at least partially suppressed with expression of S3DN.

## Discussion

Given the cumulative evidence supporting Stat3 as a potential therapeutic target in cancer and that there are ongoing clinical trials testing Stat3 inhibitors [49], our group has attempted to better understand the molecular mechanisms of Stat3 activity and the role of HGF/c-met in transducing its signal. To this aim, we have used the S3DN system, which provides a workable cell culture model to study multiple aspects of Stat3 function in detail. Here we successfully demonstrate that even partial reduction in Stat3 activity is sufficient to restrict motility and invasion. S3DN (DN2 and DN5) cells were significantly less invasive in both *in vitro *and *in vivo *assays, and this phenotype was accompanied by marked suppression of MMP-2 and MMP-9 expression, c-met activity, as well as inhibition of Stat3/c-met interaction.

The expression of S3DN reduced HGF-induced scattering and invasion through Matrigel-coated membranes (Figure 1). This assay indicates that the motile phenotype of the P9WT cells in this context is cytokine dependent and mediated by Stat3, which is relevant for tumors growing *in vivo *where they respond to a variety of cytokines and growth factors from host cells or from autocrine stimulation. The lack of complete suppression of invasiveness for the S3DN tumors indicates less than full penetrance for the effect of S3DN expression. This can be attributed to the fact that there is still active Stat3 in the S3DN cells (Figure 4, data not shown). It is also possible that the percentage of attached and invasive tumors would have been even higher for the WT and NEO4 groups had the experiment been carried out for a longer time, since it is likely that those tumors designated as attached would have eventually become invasive if allowed to grow longer.

We originally hypothesized that the mechanism for the reduced invasive behavior of S3DN cells is due to a suppression of c-met-mediated events. c-met is frequently constitutively active in human tumors [36-38], and several reports have shown that Stat3 is one of the downstream effectors of HGF/c-met signaling [20,39]. HGF treatment enhances Stat3 activity, likely due to an interaction between c-met and Stat3 [39]. We show that expression of S3DN leads to reduced baseline as well as inducible c-met activity in S3DN cells and tumors (Figure 2, 4). Inhibition of c-met activity in S3DN cells is also associated with reduced localization of p-c-met to focal adhesions (Figure 2). These findings provide a strong indication for a positive feedback loop involving HGF, c-met, and Stat3. The existence of such a feedback loop is further supported by our finding that S3DN cells are less responsive to HGF-induced motility. An analysis of the levels of endogenous HGF secretion in S3DN cells by ELISA revealed no detectable difference between the WT, NEO4 and S3DN cell lines (data not shown). However, the levels of secreted HGF in these cells were difficult to compare since they were below the detection threshold of the ELISA (< 40 pg/ml). We note that based on numerous reports concerning the biological effects of HGF, which typically fall in the ED50 range of 20-100 ng/ml, we do not expect that these cells produce sufficient amounts of HGF to ellicit an autocrine response. Our initial hypothesis was supported by our finding that there was an interaction between Stat3 and c-met proteins, and that this interaction is reduced in cells that express S3DN (Figure 6). It is possible that Stat3/c-met interaction occurs through FAK, since there are known associations between c-met and FAK [50] and Stat3 and FAK [51]. We did find an association between c-met and FAK in IP experiments, but there was no detectable difference in the degree of this interaction between WT and S3DN cells (data not shown). We speculate that S3DN is preventing the activation of c-met at the cell membrane by interfering with normal Stat3/c-met interaction, possibly by competing with Stat3 for binding to c-met.

Interestingly, we observed membrane localization of p-Stat3 (Tyr705) in WT and NEO4 tumors, but not in the S3DN tumors, which had only nuclear staining (Figure 4A). This is in line with our previously published results with human SCC samples where we observed p-Stat3 (Tyr705) staining at the cell membrane and nuclei in tumors, but only in nuclei in the immediately adjacent non-malignant skin [48]. It is possible that the membrane localization of p-Stat3 (Tyr705) is specific to cells that are more malignant. Recent published reports indicate that there are potential non-nuclear functions for Stat3. The functional flexibility of Stat3 was outlined in a review published by Gao and Bromberg [52], specifically describing a non-transcriptional, cytoplasmic role that may potentiate cell motility. A Stat3-stathmin interaction has been documented [53], as well as Stat3 localization at focal adhesions with FAK and paxillin [51]. A recent publication describes a metabolic function in the mitochondria that supports Ras-dependent transformation [54] and cellular respiration [55]. These reports indicate non-nuclear roles for Stat3 that contribute to the oncogenic properties of cancer cells and that these functions are separate from tyrosine-phosphorylation dependent transcriptional activity. We provide further support for a novel function for Stat3 by describing invasive tumors with a distinct Stat3 membrane localization. Although we do see suppression of MMP-2 and MMP-9 expression, which is likely due to transcriptional downregulation caused by S3DN expression, we propose that Stat3 may also be acting at the membrane to enhance motility and invasion (Figure 6). We further speculate that the Stat3 protein engaged in the non-transcriptional function at the membrane may be exerting a fast-acting response to external activators, whereas the transcriptional activation of genes like the MMPs by nuclear Stat3 could be a slower mechanism to potentiate cell motility. Thus, it is possible that the transcriptional and non-transcriptional functions of Stat3 are working in concert to influence cell migration, especially in malignant, invasive cells.

The lack of diminished phospho-Stat3 staining in the S3DN cells appears at first contradictory to previous data showing a positive correlation between Stat3 phosphorylation and activity. However we suggest that the S3DN protein blocks endogenous Stat3 activity at a point downstream of its phosphorylation. We have previously shown that Stat3 DNA binding, as measured by EMSA, is reduced in the S3DN cells compared to WT cells, confirming that S3DN expression has a suppressive effect on Stat3 activity [43].

Our observation that S3DN tumors grew to similar size as WT and NEO4 tumors was unexpected. A previous report, using HT-29 colon carcinoma cells in mouse xenograft experiments, showed a reduction in tumor size for cells stably expressing a similar Stat3 dominant negative form compared to parental HT-29 tumors [56]. One possible explanation for this discrepency could be the fact that the parental HT-29 cells, unlike the SRB12-p9 cells, were negative for constitutive activation of Stat3 in the absence of exogenously added growth factors. From our initial studies of the S3DN cells we observed that the proliferation rate was unaffected by S3DN expression *in vitro*, while sensitivity to exogenous growth factor deprivation was increased [43]. We hypothesized that the partial suppression of Stat3 activity in the S3DN cells was sufficient to affect survival under growth factor deprived conditions, but not sufficient to affect proliferation rate. In the case of HT-29 cells, where dominant negative Stat3 expression appears to completely block Stat3 activity[56], the tumor size is reduced even though host supplied growth factors are present. To observe a similar effect in our system would likely require stronger suppression of Stat3 activity.

## Conclusions

We have shown for the first time in an *in vivo *tumor model that the HGF/c-met/Stat3 signaling cascade is critically involved in the motility and invasion of cancer cells, and disruption of this pathway by expression of S3DN suppresses invasion, reduces c-met activity and inhibits c-met/Stat3 interaction. Tumor cells producing high levels of HGF can activate c-met by either autocrine or paracrine signaling mechanisms, which leads to Stat3 activation and gene transcription. Based on the indications for a positive feedback loop between c-met and Stat3, we speculate that abnormally high levels of c-met activation could trigger this loop and drive cells toward invasive behavior. We also show that there is a possible role for Stat3 at the cell membrane that contributes to the malignant phenotype of invading tumor cells. We must express a high degree of caution in generalizing these results to all tumor cell types or even to other skin SCC cells, since these findings are confined to a single cell line. It will be necessary to perform a similar series of experiments on other skin SCC cell lines, as well as other tumor cell types. Attempts to generate additional stably expressing S3DN cells using other skin-derived cell lines, and cell lines of other tumor types, have been unsuccessful so far. This is perhaps due to a S3DN-mediated suppression of proliferation and/or survival that is too strong in those cells. The SRB12-p9 cells are likely to be unique in having a very strong constitutive Stat3 activity when grown in the presence of serum, thereby allowing survival of stable S3DN clones. Never-the-less, based on these results we suggest that targeting the HGF/c-met/Stat3 pathway could be an especially effective strategy for cancer therapy. Future work will focus on exploring the interaction between Stat3 and c-met, and the relationship of the HGF/c-met/Stat3 signaling loop to the invasive potential of skin SCC cells.

## Competing interests

The authors declare that they have no competing interests.

## Authors' contributions

ZAS carried out *in vitro *invasion, motility, adhesion, and soft agar assays, immunohistochemistry, Western blotting, and contributed to the draft of the manuscript. WY generated and characterized the S3DN cell lines. KH assisted with the immunohistochemistry, Western blotting, and *in vitro *cell assays. JNG maintained the mice, assisted with tumor harvest, and immunohistochemistry. RS performed statistical analysis for the design and interpretation of the mouse tumor Xenograft experiments. JLC contributed to the conception and design of the study, coordinated the study, contributed to the microscopy, tissue harvesting, and final editing of the draft of the manuscript. All authors have read and approved the final manuscript.

## Pre-publication history

The pre-publication history for this paper can be accessed here:

http://www.biomedcentral.com/1471-2407/11/180/prepub
